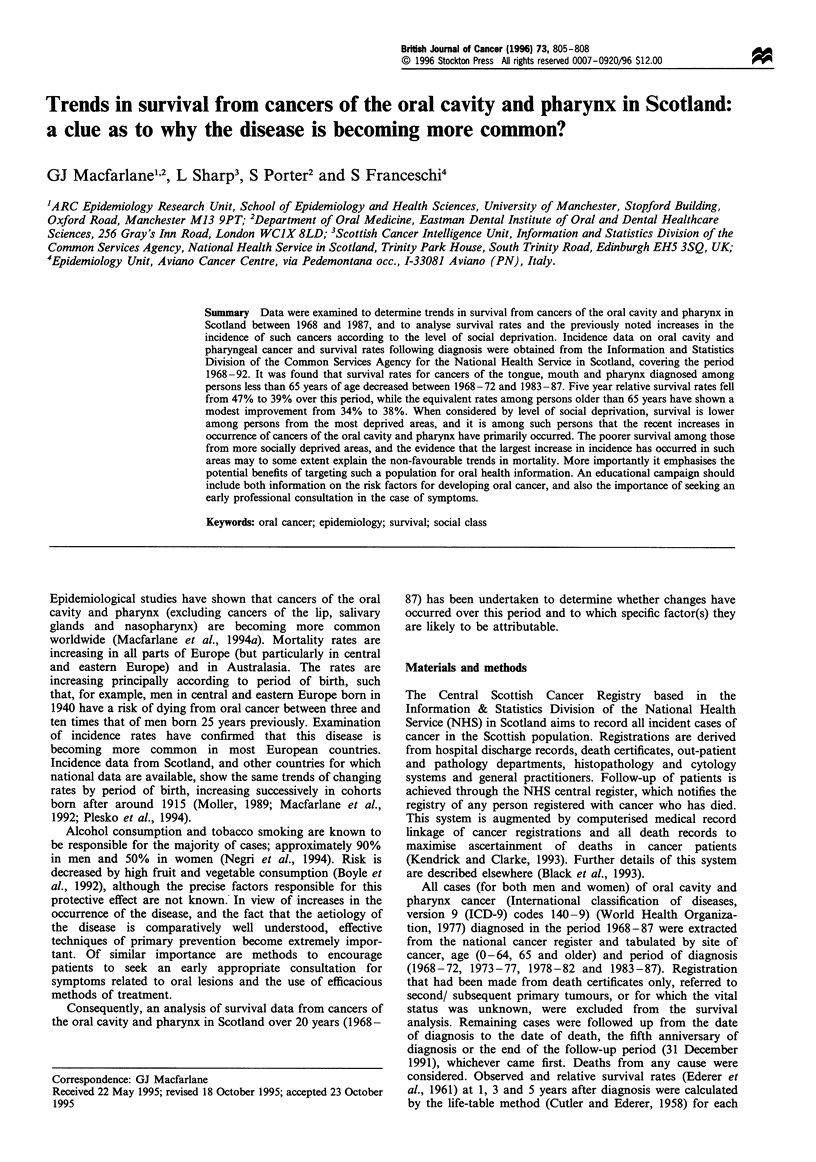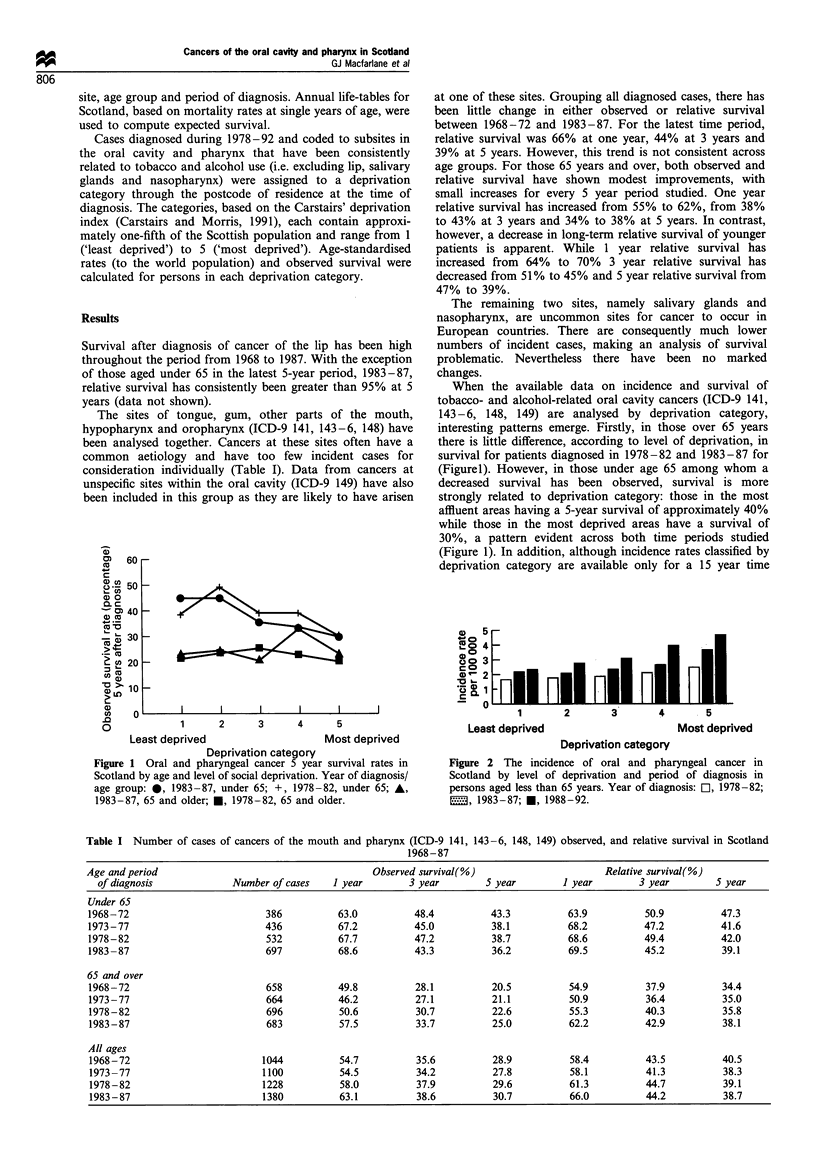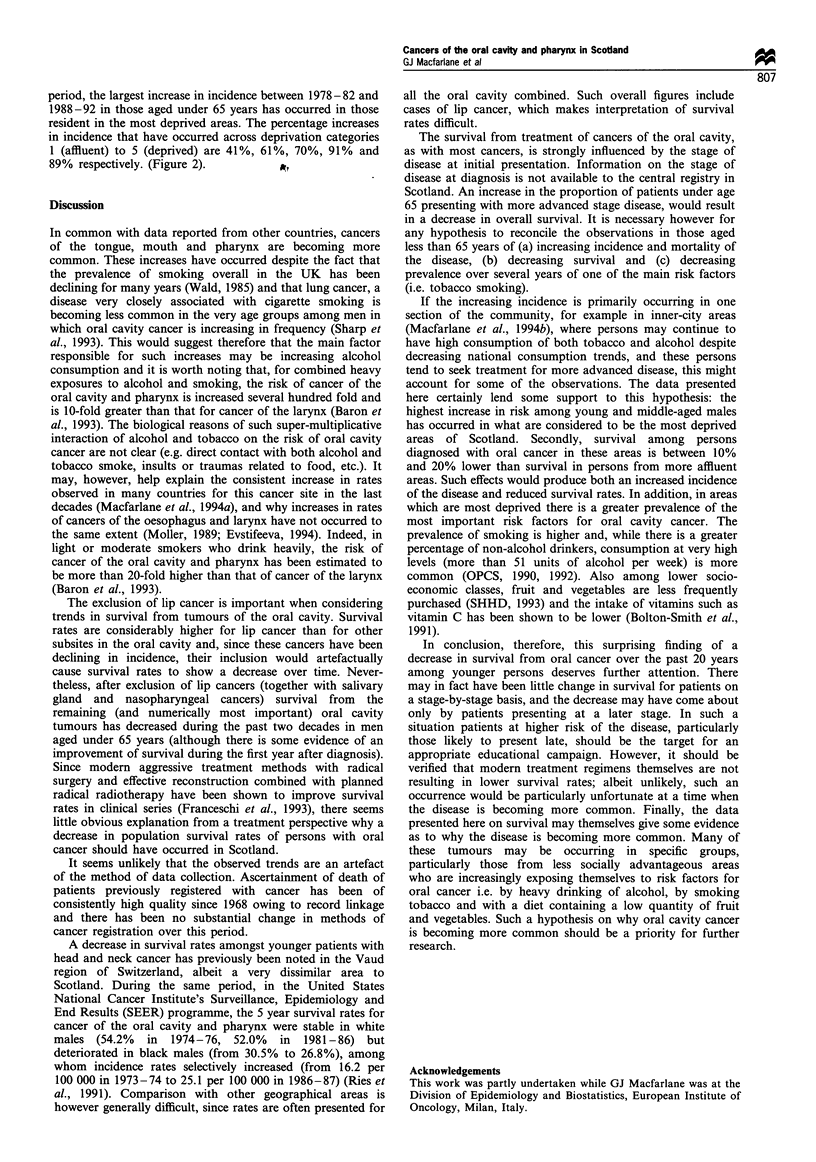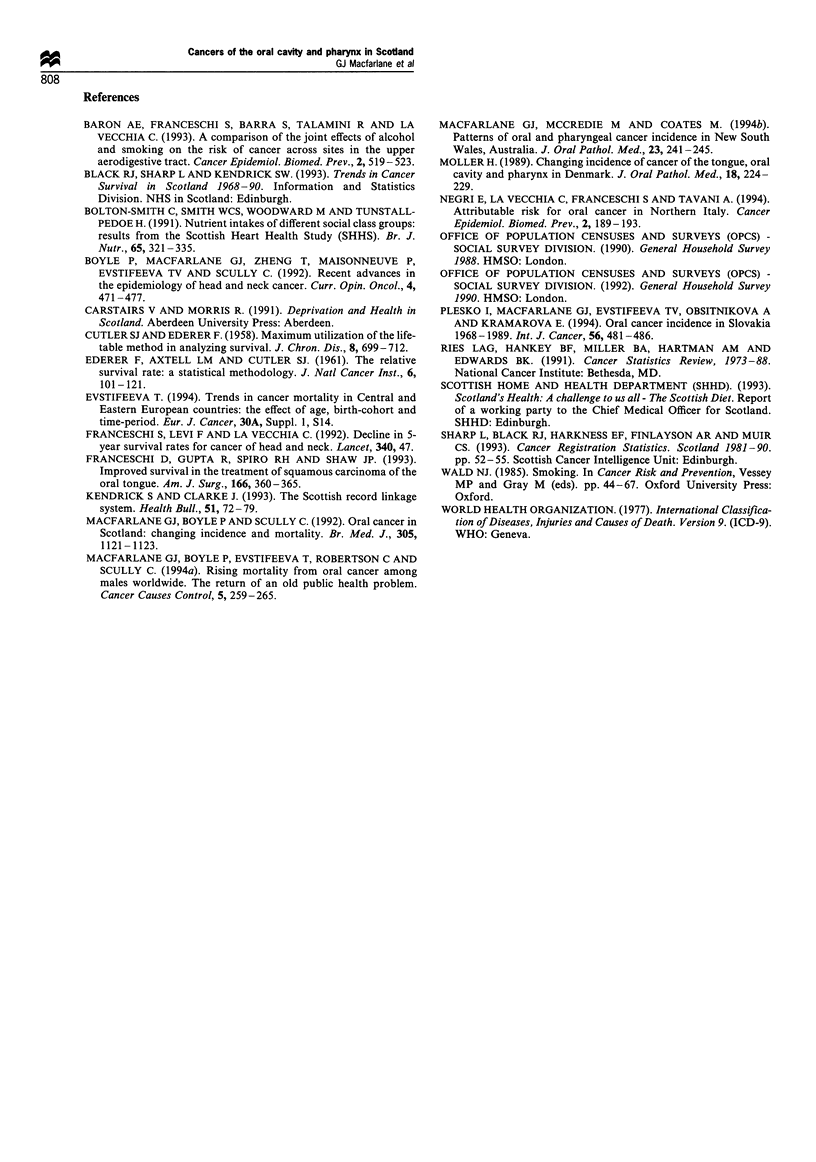# Trends in survival from cancers of the oral cavity and pharynx in Scotland: a clue as to why the disease is becoming more common?

**DOI:** 10.1038/bjc.1996.141

**Published:** 1996-03

**Authors:** G. J. Macfarlane, L. Sharp, S. Porter, S. Franceschi

**Affiliations:** ARC Epidemiology Research Unit, School of Epidemiology and Health Sciences, University of Manchester, UK.

## Abstract

Data were examined to determine trends in survival from cancers of the oral cavity and pharynx in Scotland between 1968 and 1987, and to analyse survival rates and the previously noted increases in the incidence of such cancers according to the level of social deprivation. Incidence data on oral cavity and pharyngeal cancer and survival rates following diagnosis were obtained from the Information and Statistics Division of the Common Services Agency for the National Health Service in Scotland, covering the period 1968-92. It was found that survival rates for cancers of the tongue, mouth and pharynx diagnosed among persons less than 65 years of age decreased between 1968-72 and 1983-87. Five year relative survival rates fell from 47% to 39% over this period, while the equivalent rates among persons older than 65 years have shown a modest improvement from 34% to 38%. When considered by level of social deprivation, survival is lower among persons from the most deprived areas, and it is among such persons that the recent increases in occurrence of cancers of the oral cavity and pharynx have primarily occurred. The poorer survival among those from more socially deprived areas, and the evidence that the largest increase in incidence has occurred in such areas may to some extent explain the non-favourable trends in mortality. More importantly it emphasises the potential benefits of targeting such a population for oral health information. An educational campaign should include both information on the risk factors for developing oral cancer, and also the importance of seeking an early professional consultation in the case of symptoms.


British Journal of Cancer (1996) 73, 805-808
© 1996 Stockton Press All rights reserved 0007-0920/96 $12.00

### Trends in survival from cancers of the oral cavity and pharynx in Scotland: a clue as to why the disease is becoming more common?

GJ Macfarlane[1,2], L Sharp[3], S Porter[2] and S Franceschi[4]

[1]*ARC Epidemiology Research Unit, School of Epidemiology and Health Sciences, University of Manchester, Stopford Building, Oxford Road, Manchester M13 9PT;* [2]*Department of Oral Medicine, Eastman Dental Institute of Oral and Dental Healthcare Sciences, 256 Gray's Inn Road, London WC1X 8LD;* [3]*Scottish Cancer Intelligence Unit, Information and Statistics Division of the Common Services Agency, National Health Service in Scotland, Trinity Park House, South Trinity Road, Edinburgh EH5 3SQ, UK;* [4]*Epidemiology Unit, Aviano Cancer Centre, via Pedemontana occ., I-33081 Aviano (PN), Italy.*

**Summary** Data were examined to determine trends in survival from cancers of the oral cavity and pharynx in Scotland between 1968 and 1987, and to analyse survival rates and the previously noted increases in the incidence of such cancers according to the level of social deprivation. Incidence data on oral cavity and pharyngeal cancer and survival rates following diagnosis were obtained from the Information and Statistics Division of the Common Services Agency for the National Health Service in Scotland, covering the period 1968-92. It was found that survival rates for cancers of the tongue, mouth and pharynx diagnosed among persons less than 65 years of age decreased between 1968-72 and 1983-87. Five year relative survival rates fell from 47% to 39% over this period, while the equivalent rates among persons older than 65 years have shown a modest improvement from 34% to 38%. When considered by level of social deprivation, survival is lower among persons from the most deprived areas, and it is among such persons that the recent increases in occurrence of cancers of the oral cavity and pharynx have primarily occurred. The poorer survival among those from more socially deprived areas, and the evidence that the largest increase in incidence has occurred in such areas may to some extent explain the non-favourable trends in mortality. More importantly it emphasises the potential benefits of targeting such a population for oral health information. An educational campaign should include both information on the risk factors for developing oral cancer, and also the importance of seeking an early professional consultation in the case of symptoms.

**Keywords:** oral cancer; epidemiology; survival; social class

Epidemiological studies have shown that cancers of the oral cavity and pharynx (excluding cancers of the lip, salivary glands and nasopharynx) are becoming more common worldwide (Macfarlane *et al.*, 1994*a*). Mortality rates are increasing in all parts of Europe (but particularly in central and eastern Europe) and in Australasia. The rates are increasing principally according to period of birth, such that, for example, men in central and eastern Europe born in 1940 have a risk of dying from oral cancer between three and ten times that of men born 25 years previously. Examination of incidence rates have confirmed that this disease is becoming more common in most European countries. Incidence data from Scotland, and other countries for which national data are available, show the same trends of changing rates by period of birth, increasing successively in cohorts born after around 1915 (Moller, 1989; Macfarlane *et al.*, 1992; Plesko *et al.*, 1994).

Alcohol consumption and tobacco smoking are known to be responsible for the majority of cases; approximately 90% in men and 50% in women (Negri *et al.*, 1994). Risk is decreased by high fruit and vegetable consumption (Boyle *et al.*, 1992), although the precise factors responsible for this protective effect are not known. In view of increases in the occurrence of the disease, and the fact that the aetiology of the disease is comparatively well understood, effective techniques of primary prevention become extremely important. Of similar importance are methods to encourage patients to seek an early appropriate consultation for symptoms related to oral lesions and the use of efficacious methods of treatment.

Consequently, an analysis of survival data from cancers of the oral cavity and pharynx in Scotland over 20 years (1968-87) has been undertaken to determine whether changes have occurred over this period and to which specific factor(s) they are likely to be attributable.

Correspondence: GJ Macfarlane
Received 22 May 1995; revised 18 October 1995; accepted 23 October 1995

### Materials and methods

The Central Scottish Cancer Registry based in the Information & Statistics Division of the National Health Service (NHS) in Scotland aims to record all incident cases of cancer in the Scottish population. Registrations are derived from hospital discharge records, death certificates, out-patient and pathology departments, histopathology and cytology systems and general practitioners. Follow-up of patients is achieved through the NHS central register, which notifies the registry of any person registered with cancer who has died. This system is augmented by computerised medical record linkage of cancer registrations and all death records to maximise ascertainment of deaths in cancer patients (Kendrick and Clarke, 1993). Further details of this system are described elsewhere (Black *et al.*, 1993).

All cases (for both men and women) of oral cavity and pharynx cancer (International classification of diseases, version 9 (ICD-9) codes 140-9) (World Health Organization, 1977) diagnosed in the period 1968-87 were extracted from the national cancer register and tabulated by site of cancer, age (0-64, 65 and older) and period of diagnosis (1968-72, 1973-77, 1978-82 and 1983-87). Registration that had been made from death certificates only, referred to second/ subsequent primary tumours, or for which the vital status was unknown, were excluded from the survival analysis. Remaining cases were followed up from the date of diagnosis to the date of death, the fifth anniversary of diagnosis or the end of the follow-up period (31 December 1991), whichever came first. Deaths from any cause were considered. Observed and relative survival rates (Ederer *et al.*, 1961) at 1, 3 and 5 years after diagnosis were calculated by the life-table method (Cutler and Ederer, 1958) for each site, age group and period of diagnosis. Annual life-tables for Scotland, based on mortality rates at single years of age, were used to compute expected survival.

Cases diagnosed during 1978–92 and coded to subsites in the oral cavity and pharynx that have been consistently related to tobacco and alcohol use (i.e. excluding lip, salivary glands and nasopharynx) were assigned to a deprivation category through the postcode of residence at the time of diagnosis. The categories, based on the Carstairs' deprivation index (Carstairs and Morris, 1991), each contain approximately one-fifth of the Scottish population and range from 1 ('least deprived') to 5 ('most deprived'). Age-standardised rates (to the world population) and observed survival were calculated for persons in each deprivation category.

### Results

Survival after diagnosis of cancer of the lip has been high throughout the period from 1968 to 1987. With the exception of those aged under 65 in the latest 5-year period, 1983–87, relative survival has consistently been greater than 95% at 5 years (data not shown).

The sites of tongue, gum, other parts of the mouth, hypopharynx and oropharynx (ICD-9 141, 143–6, 148) have been analysed together. Cancers at these sites often have a common aetiology and have too few incident cases for consideration individually (Table I). Data from cancers at unspecific sites within the oral cavity (ICD-9 149) have also been included in this group as they are likely to have arisen at one of these sites. Grouping all diagnosed cases, there has been little change in either observed or relative survival between 1968–72 and 1983–87. For the latest time period, relative survival was 66% at one year, 44% at 3 years and 39% at 5 years. However, this trend is not consistent across age groups. For those 65 years and over, both observed and relative survival have shown modest improvements, with small increases for every 5 year period studied. One year relative survival has increased from 55% to 62%, from 38% to 43% at 3 years and 34% to 38% at 5 years. In contrast, however, a decrease in long-term relative survival of younger patients is apparent. While 1 year relative survival has increased from 64% to 70% 3 year relative survival has decreased from 51% to 45% and 5 year relative survival from 47% to 39%.

The remaining two sites, namely salivary glands and nasopharynx, are uncommon sites for cancer to occur in European countries. There are consequently much lower numbers of incident cases, making an analysis of survival problematic. Nevertheless there have been no marked changes.

When the available data on incidence and survival of tobacco- and alcohol-related oral cavity cancers (ICD-9 141, 143–6, 148, 149) are analysed by deprivation category, interesting patterns emerge. Firstly, in those over 65 years there is little difference, according to level of deprivation, in survival for patients diagnosed in 1978–82 and 1983–87 for (Figurel). However, in those under age 65 among whom a decreased survival has been observed, survival is more strongly related to deprivation category: those in the most affluent areas having a 5-year survival of approximately 40% while those in the most deprived areas have a survival of 30%, a pattern evident across both time periods studied (Figure 1). In addition, although incidence rates classified by deprivation category are available only for a 15 year time

**Figure 1** Oral and pharyngeal cancer 5 year survival rates in Scotland by age and level of social deprivation. Year of diagnosis/ age group: ●, 1983–87, under 65; +, 1978–82, under 65; ▲, 1983–87, 65 and older; ■, 1978–82, 65 and older.

**Figure 2** The incidence of oral and pharyngeal cancer in Scotland by level of deprivation and period of diagnosis in persons aged less than 65 years. Year of diagnosis: □, 1978–82; ▦, 1983–87; ■, 1988–92.

**Table I** Number of cases of cancers of the mouth and pharynx (ICD-9 141, 143–6, 148, 149) observed, and relative survival in Scotland 1968–87

| *Age and period of diagnosis* | *Number of cases* | *Observed survival(%)* 1 year | 3 year | 5 year | *Relative survival(%)* 1 year | 3 year | 5 year |
|---|---|---|---|---|---|---|---|
| *Under 65* | | | | | | | |
| 1968–72 | 386 | 63.0 | 48.4 | 43.3 | 63.9 | 50.9 | 47.3 |
| 1973–77 | 436 | 67.2 | 45.0 | 38.1 | 68.2 | 47.2 | 41.6 |
| 1978–82 | 532 | 67.7 | 47.2 | 38.7 | 68.6 | 49.4 | 42.0 |
| 1983–87 | 697 | 68.6 | 43.3 | 36.2 | 69.5 | 45.2 | 39.1 |
| *65 and over* | | | | | | | |
| 1968–72 | 658 | 49.8 | 28.1 | 20.5 | 54.9 | 37.9 | 34.4 |
| 1973–77 | 664 | 46.2 | 27.1 | 21.1 | 50.9 | 36.4 | 35.0 |
| 1978–82 | 696 | 50.6 | 30.7 | 22.6 | 55.3 | 40.3 | 35.8 |
| 1983–87 | 683 | 57.5 | 33.7 | 25.0 | 62.2 | 42.9 | 38.1 |
| *All ages* | | | | | | | |
| 1968–72 | 1044 | 54.7 | 35.6 | 28.9 | 58.4 | 43.5 | 40.5 |
| 1973–77 | 1100 | 54.5 | 34.2 | 27.8 | 58.1 | 41.3 | 38.3 |
| 1978–82 | 1228 | 58.0 | 37.9 | 29.6 | 61.3 | 44.7 | 39.1 |
| 1983–87 | 1380 | 63.1 | 38.6 | 30.7 | 66.0 | 44.2 | 38.7 |

period, the largest increase in incidence between 1978-82 and 1988-92 in those aged under 65 years has occurred in those resident in the most deprived areas. The percentage increases in incidence that have occurred across deprivation categories 1 (affluent) to 5 (deprived) are 41%, 61%, 70%, 91% and 89% respectively. (Figure 2).

### Discussion

In common with data reported from other countries, cancers of the tongue, mouth and pharynx are becoming more common. These increases have occurred despite the fact that the prevalence of smoking overall in the UK has been declining for many years (Wald, 1985) and that lung cancer, a disease very closely associated with cigarette smoking is becoming less common in the very age groups among men in which oral cavity cancer is increasing in frequency (Sharp *et al.*, 1993). This would suggest therefore that the main factor responsible for such increases may be increasing alcohol consumption and it is worth noting that, for combined heavy exposures to alcohol and smoking, the risk of cancer of the oral cavity and pharynx is increased several hundred fold and is 10-fold greater than that for cancer of the larynx (Baron *et al.*, 1993). The biological reasons of such super-multiplicative interaction of alcohol and tobacco on the risk of oral cavity cancer are not clear (e.g. direct contact with both alcohol and tobacco smoke, insults or traumas related to food, etc.). It may, however, help explain the consistent increase in rates observed in many countries for this cancer site in the last decades (Macfarlane *et al.*, 1994*a*), and why increases in rates of cancers of the oesophagus and larynx have not occurred to the same extent (Moller, 1989; Evstifeeva, 1994). Indeed, in light or moderate smokers who drink heavily, the risk of cancer of the oral cavity and pharynx has been estimated to be more than 20-fold higher than that of cancer of the larynx (Baron *et al.*, 1993).

The exclusion of lip cancer is important when considering trends in survival from tumours of the oral cavity. Survival rates are considerably higher for lip cancer than for other subsites in the oral cavity and, since these cancers have been declining in incidence, their inclusion would artefactually cause survival rates to show a decrease over time. Nevertheless, after exclusion of lip cancers (together with salivary gland and nasopharyngeal cancers) survival from the remaining (and numerically most important) oral cavity tumours has decreased during the past two decades in men aged under 65 years (although there is some evidence of an improvement of survival during the first year after diagnosis). Since modern aggressive treatment methods with radical surgery and effective reconstruction combined with planned radical radiotherapy have been shown to improve survival rates in clinical series (Franceschi *et al.*, 1993), there seems little obvious explanation from a treatment perspective why a decrease in population survival rates of persons with oral cancer should have occurred in Scotland.

It seems unlikely that the observed trends are an artefact of the method of data collection. Ascertainment of death of patients previously registered with cancer has been of consistently high quality since 1968 owing to record linkage and there has been no substantial change in methods of cancer registration over this period.

A decrease in survival rates amongst younger patients with head and neck cancer has previously been noted in the Vaud region of Switzerland, albeit a very dissimilar area to Scotland. During the same period, in the United States National Cancer Institute's Surveillance, Epidemiology and End Results (SEER) programme, the 5 year survival rates for cancer of the oral cavity and pharynx were stable in white males (54.2% in 1974-76, 52.0% in 1981-86) but deteriorated in black males (from 30.5% to 26.8%), among whom incidence rates selectively increased (from 16.2 per 100 000 in 1973-74 to 25.1 per 100 000 in 1986-87) (Ries *et al.*, 1991). Comparison with other geographical areas is however generally difficult, since rates are often presented for all the oral cavity combined. Such overall figures include cases of lip cancer, which makes interpretation of survival rates difficult.

The survival from treatment of cancers of the oral cavity, as with most cancers, is strongly influenced by the stage of disease at initial presentation. Information on the stage of disease at diagnosis is not available to the central registry in Scotland. An increase in the proportion of patients under age 65 presenting with more advanced stage disease, would result in a decrease in overall survival. It is necessary however for any hypothesis to reconcile the observations in those aged less than 65 years of (a) increasing incidence and mortality of the disease, (b) decreasing survival and (c) decreasing prevalence over several years of one of the main risk factors (i.e. tobacco smoking).

If the increasing incidence is primarily occurring in one section of the community, for example in inner-city areas (Macfarlane *et al.*, 1994*b*), where persons may continue to have high consumption of both tobacco and alcohol despite decreasing national consumption trends, and these persons tend to seek treatment for more advanced disease, this might account for some of the observations. The data presented here certainly lend some support to this hypothesis: the highest increase in risk among young and middle-aged males has occurred in what are considered to be the most deprived areas of Scotland. Secondly, survival among persons diagnosed with oral cancer in these areas is between 10% and 20% lower than survival in persons from more affluent areas. Such effects would produce both an increased incidence of the disease and reduced survival rates. In addition, in areas which are most deprived there is a greater prevalence of the most important risk factors for oral cavity cancer. The prevalence of smoking is higher and, while there is a greater percentage of non-alcohol drinkers, consumption at very high levels (more than 51 units of alcohol per week) is more common (OPCS, 1990, 1992). Also among lower socio-economic classes, fruit and vegetables are less frequently purchased (SHHD, 1993) and the intake of vitamins such as vitamin C has been shown to be lower (Bolton-Smith *et al.*, 1991).

In conclusion, therefore, this surprising finding of a decrease in survival from oral cancer over the past 20 years among younger persons deserves further attention. There may in fact have been little change in survival for patients on a stage-by-stage basis, and the decrease may have come about only by patients presenting at a later stage. In such a situation patients at higher risk of the disease, particularly those likely to present late, should be the target for an appropriate educational campaign. However, it should be verified that modern treatment regimens themselves are not resulting in lower survival rates; albeit unlikely, such an occurrence would be particularly unfortunate at a time when the disease is becoming more common. Finally, the data presented here on survival may themselves give some evidence as to why the disease is becoming more common. Many of these tumours may be occurring in specific groups, particularly those from less socially advantageous areas who are increasingly exposing themselves to risk factors for oral cancer i.e. by heavy drinking of alcohol, by smoking tobacco and with a diet containing a low quantity of fruit and vegetables. Such a hypothesis on why oral cavity cancer is becoming more common should be a priority for further research.

**Acknowledgements**
This work was partly undertaken while GJ Macfarlane was at the Division of Epidemiology and Biostatistics, European Institute of Oncology, Milan, Italy.

### References

BARON AE, FRANCESCHI S, BARRA S, TALAMINI R AND LA VECCHIA C. (1993). A comparison of the joint effects of alcohol and smoking on the risk of cancer across sites in the upper aerodigestive tract. *Cancer Epidemiol. Biomed. Prev.*, **2,** 519-523.

BLACK RJ, SHARP L AND KENDRICK SW. (1993). *Trends in Cancer Survival in Scotland 1968-90.* Information and Statistics Division. NHS in Scotland: Edinburgh.

BOLTON-SMITH C, SMITH WCS, WOODWARD M AND TUNSTALL-PEDOE H. (1991). Nutrient intakes of different social class groups: results from the Scottish Heart Health Study (SHHS). *Br. J. Nutr.*, **65,** 321-335.

BOYLE P, MACFARLANE GJ, ZHENG T, MAISONNEUVE P, EVSTIFEEVA TV AND SCULLY C. (1992). Recent advances in the epidemiology of head and neck cancer. *Curr. Opin. Oncol.*, **4,** 471-477.

CARSTAIRS V AND MORRIS R. (1991). *Deprivation and Health in Scotland.* Aberdeen University Press: Aberdeen.

CUTLER SJ AND EDERER F. (1958). Maximum utilization of the life-table method in analyzing survival. *J. Chron. Dis.*, **8,** 699-712.

EDERER F, AXTELL LM AND CUTLER SJ. (1961). The relative survival rate: a statistical methodology. *J. Natl Cancer Inst.*, **6,** 101-121.

EVSTIFEEVA T. (1994). Trends in cancer mortality in Central and Eastern European countries: the effect of age, birth-cohort and time-period. *Eur. J. Cancer*, **30A,** Suppl. 1, S14.

FRANCESCHI S, LEVI F AND LA VECCHIA C. (1992). Decline in 5-year survival rates for cancer of head and neck. *Lancet*, **340,** 47.

FRANCESCHI D, GUPTA R, SPIRO RH AND SHAW JP. (1993). Improved survival in the treatment of squamous carcinoma of the oral tongue. *Am. J. Surg.*, **166,** 360-365.

KENDRICK S AND CLARKE J. (1993). The Scottish record linkage system. *Health Bull.*, **51,** 72-79.

MACFARLANE GJ, BOYLE P AND SCULLY C. (1992). Oral cancer in Scotland: changing incidence and mortality. *Br. Med. J.*, **305,** 1121-1123.

MACFARLANE GJ, BOYLE P, EVSTIFEEVA T, ROBERTSON C AND SCULLY C. (1994*a*). Rising mortality from oral cancer among males worldwide. The return of an old public health problem. *Cancer Causes Control*, **5,** 259-265.

MACFARLANE GJ, MCCREDIE M AND COATES M. (1994*b*). Patterns of oral and pharyngeal cancer incidence in New South Wales, Australia. *J. Oral Pathol. Med.*, **23,** 241-245.

MOLLER H. (1989). Changing incidence of cancer of the tongue, oral cavity and pharynx in Denmark. *J. Oral Pathol. Med.*, **18,** 224-229.

NEGRI E, LA VECCHIA C, FRANCESCHI S AND TAVANI A. (1994). Attributable risk for oral cancer in Northern Italy. *Cancer Epidemiol. Biomed. Prev.*, **2,** 189-193.

OFFICE OF POPULATION CENSUSES AND SURVEYS (OPCS) - SOCIAL SURVEY DIVISION. (1990). *General Household Survey 1988.* HMSO: London.

OFFICE OF POPULATION CENSUSES AND SURVEYS (OPCS) - SOCIAL SURVEY DIVISION. (1992). *General Household Survey 1990.* HMSO: London.

PLESKO I, MACFARLANE GJ, EVSTIFEEVA TV, OBSITNIKOVA A AND KRAMAROVA E. (1994). Oral cancer incidence in Slovakia 1968-1989. *Int. J. Cancer*, **56,** 481-486.

RIES LAG, HANKEY BF, MILLER BA, HARTMAN AM AND EDWARDS BK. (1991). *Cancer Statistics Review, 1973-88.* National Cancer Institute: Bethesda, MD.

SCOTTISH HOME AND HEALTH DEPARTMENT (SHHD). (1993). *Scotland's Health: A challenge to us all - The Scottish Diet.* Report of a working party to the Chief Medical Officer for Scotland. SHHD: Edinburgh.

SHARP L, BLACK RJ, HARKNESS EF, FINLAYSON AR AND MUIR CS. (1993). *Cancer Registration Statistics. Scotland 1981-90.* pp. 52-55. Scottish Cancer Intelligence Unit: Edinburgh.

WALD NJ. (1985). Smoking. In *Cancer Risk and Prevention*, Vessey MP and Gray M (eds). pp. 44-67. Oxford University Press: Oxford.

WORLD HEALTH ORGANIZATION. (1977). *International Classification of Diseases, Injuries and Causes of Death. Version 9.* (ICD-9). WHO: Geneva.